# Injectable organic and inorganic selenium in dairy cows – Effects on milk, blood and somatic cell count levels

**DOI:** 10.4102/ojvr.v86i1.1664

**Published:** 2019-10-08

**Authors:** Gert M. Ferreira, Inge-Marie Petzer

**Affiliations:** 1 Morvet Veterinary Practise, Potchefstroom, South Africa; 2 Department of Production Animal Studies, Faculty of Veterinary Science, University of Pretoria, Pretoria, South Africa

**Keywords:** dairy cows, injectable selenium, Na-selenite, Se-methionine, serum selenium, milk selenium, SCC, somatic cell count

## Abstract

Mastitis is the most costly disease of dairy cows. A pro-active approach includes insuring adequate levels of selective trace minerals. The aim of this study was to determine the effect of two different commercially available, injectable selenium products, (sodium) Na-selenite (inorganic) and (selenium) Se-methionine (organic), on milk composition and on serum and milk selenium concentrations in high-yielding Holstein cows on total mix ration. Sixty multiparous cows were randomly selected into three groups of 20, one control group and two groups supplemented with injectable trace minerals. Blood and milk samples were collected over a period of 60 days. No specific change was indicated in milk yield, lactose, milk urea nitrogen (MUN) and milk pH levels compared with baseline values. The Se-methionine supplemented group showed a numerical increase in total milk protein percentage. In the group injected with Se-methionine, a negative correlation was present for the initial 72 hours between serum selenium concentration and somatic cell count (SCC) and a highly significant (*p* < 0.001) increase in milk selenium concentration for the initial 24 hours. Serum selenium concentration of Se-methionine-supplemented cows was however not significantly changed. Injection of Na-selenite led to a 60-day initial increase in serum selenium concentration above baseline levels and a significant milk selenium concentration on day 1 but to a negative correlation between serum selenium concentration and SCC. Differences in serum and milk selenium concentrations followed with the use of organic and inorganic selenium injectables. Injectable Na-selenite, as selenium, can be of important value for cattle farmers if supplemented on strategically physiological periods to improve production, reproduction and immunity.

## Introduction

The productive performance of dairy cattle may be influenced by several factors, but mineral imbalances are crucial in terms of direct effects on production (Juniper et al. 2006). The National Research Council of America (NRC) has identified 10 trace minerals as essential for cattle of which only 4 are generally recognised as the most problematic in grazing cattle: copper (Cu), zinc (Zn), manganese (Mn) and selenium (Se) (Aloha 2008; Oltramari et al. 2014).

Stress conditions negatively affect feed intake, metabolism, available energy and milk production, leading to lower butterfat composition (Amorla-Philips 2013). Trace minerals play an important role as co-enzymes in various metabolic processes, antioxidants and immune responses (Arthington & Havenga 2012). Minimising the effect of stress in ruminants by supporting the antioxidant defense mechanisms is designed to keep the reactive oxygen species (ROS) in balance (Celi et al. 2014). Oxidative stress occurs when the production of ROS exceeds the antioxidant defense mechanisms present in the body (Spears, William & Weiss 2008). It has been demonstrated that supplementation of trace mineral has a beneficial impact on blood values as well as on milk production, composition and udder health (Juniper et al. 2006; Slavik et al. 2008). Adequate trace mineral intake and bioavailability is also required for a variety of metabolic functions, including immune and enzyme response and reproduction (Overto and Yasui, 2014). Selenium deficiency in dairy cows reduces the ability of blood and milk neutrophils to kill bacteria (Spears et al. 2008). Selenium competes with sulfur in biochemical pathways and when incorporated into the sulfur containing amino acids (cysteine and methionine), it leads to a meaningful increase in the activity of erythrocyte glutathione peroxidase Slavik et al. (2008).

Trace mineral availability in cattle is complicated by several factors, among which are the impacts of trace mineral antagonists in grazed forage and water sources, the unpredictability of uniform intake of free-choice mineral supplements and the bioavailability of supplemented trace minerals (Juniper et al. 2006). To minimise the effect of antagonism observed with oral intake of trace minerals or the possible oxidative stress with injectable unbound elements (Shen, Yang & Ong 1999), trace minerals are being chelated. Chelation is the capture of a positive-charged metal ion by a large molecule, such as EDTA (a synthetic amino acid called ethylene diamine tetra-acetic acid) (Virbac Technical Manual).

The metabolism of orally supplemented selenium sources differ in that most of the inorganic selenium, selenate (SO_4_) transformed in the rumen to selenite (SO_3_), is used to form seleno amino acids for bacterial protein and seleno specific enzymes and the rest of the selenite is absorbed in the small intestine (Weiss 2010). Se-methionine however is absorbed in the intestine and is used to form enzymes (Weiss 2010). Mehdi and Dufrasne (2016) indicated that cows supplemented orally with organic selenium showed an increase in milk selenium concentrations of 190% higher than those that received inorganic selenium. Liu et al. (2015) found elevated selenium concentrations in cheese manufactured from milk of supplemented cows.

An advantage of injectable trace mineral is the targeted delivery of a known amount of trace mineral to animals that can be administered before critical physiological periods in the production cycle (Juniper et al. 2006). Mc Dowell et al. (2005) indicated that repeated injections with (selenium) Se-methionine can increase the milk selenium levels as much as 27–54 fold compared to no treatment.

Schwarz et al. (2010) indicated that injectable trace mineral tends to reduce somatic cell count (SCC) in milk and the proportion of cows diagnosed with subclinical mastitis, and that cure of subclinical mastitis was associated with higher serum concentrations of phosphorus and selenium.

Chronically high intakes of selenium can have negative effects on human health. The Food and Drug Administration (FDA) of the United States Department of Health and Human Services set the maximum allowable concentration of selenium in milk for human consumption at 0.14 mg/L (Weiss 2010).

The reason for evaluating different injectable selenium formulations is to compare the bioavailability of the supplemented selenium. The aim of this case-control study was to assess the effect of the two injectable trace minerals with a different selenium source, by evaluating selenium concentrations in serum and milk and the effect on milk yield and composition.

## Material and methods

### Experimental animals

Sixty multiparous high merit Holstein cows were selected, from an available 1200 animals, according to the following criteria: all cows were multiparous (2–4 parities), between 4 and 6 years old, clinical healthy, well fed and with recorded data of functional udder health and milk production records between 25 kg/day and 30 kg/day on a 3 times daily milking routine with a body condition score (BCS) of 3.5–4.0 on a scale of 1–5. They all tested negative on the prescribed tests for Brucellosis (Brucella abortus), Bovine Tuberculosis (Mycobacterium tuberculosis), Enzootic Bovine Leucosis (Bovine Leucosis Virus), Bovine Viral Diarrhoea (BVD) and Virus Persistent Infection (PI). All cows were fed twice daily on a total mix ration (TMR) of which the chemical composition and the dry matter intake was monitored by a leading feed company (Nutri-Genics, Lyttleton Manor, Pretoria, South Africa [SA]) prior to and during the trial period. The ration fed during the trial period consisted of the following: molasses syrup (75/25), maize silage (24 kg), eragrostis (3.9 kg), lusern (3.2 kg), brewers grain (7.2 kg), maroek (sorghum) (1.3 kg), dairy concentrated (10.9 kg) and a fertility pack (trace minerals, amino acids, vitamins). The selenium required is 8.55 mg and cows were orally supplemented via the dairy concentrate (as determined by the nutritionist on site) at 15.71 mg/day. The selenium diet concentration was 0.55 ppm.

### Experimental design

The cows were randomly divided into three (3) groups of twenty (20) cows per group. The three groups were randomly selected as control, treatment group T1 and treatment group T2. The ear tag number and group of each cow participating in the trial was recorded. The treatment groups (T1 and T2) were injected each with a commercial combination of trace elements and the control group (C) with saline. The two commercial products were Multimin (Inorganic Na [sodium] Selenite) (Virbac SA, Halfway House, SA) and Complex + ADE Cattle (Organic Se-methionine) (Cipla Agrimed, Momumentpark, Prertoria, SA). Blood and milk samples were analysed. The follow-up period is regarded as the period of activity of the medicine (2 months) for the targeted animals (cows).

On day 14, after recording of the animal’s identification and allocating the animals to the three different groups, all the cows were weighed and treated for internal and external parasites with Noromectin (Norbrook, Isando, Gauteng, South Africa) pour-on at 1 mL/10 kg, with zero milk withdraw and kept on the same (existing) TMR for the duration (60 days) of the trial. On day zero, all animals were weighed again, clinically evaluated by a veterinarian and treated according to the protocol. All the cows from the three different groups were put together into one camp with enough trough space for food and water.

Treatment group T1 (Na-selenite) was injected with a commercially available combination of trace elements (Multimin Se + Cu for Cattle from Virbac SA) according to the recommended dose of 1 mL/100 kg. The selenium is supplemented as Na-selenite (inorganic) and the Cu, Mn and Zn as EDTA chelates. The animals, together with the Multimin, were also injected with vitamin A and E (Pro-Vit A from Virbac SA) at a dose of 1 mL/250 kg. Animals in treatment group T2 (Se-methionine) were injected with a commercially available combination of trace elements and vitamin ADE (Complex + ADE for Cattle from Cipla SA) (Cipla Technical Manual) according to the recommended dose of 1 mL/50 kg. The selenium is supplemented as Se-methionine (organic) and the Cu, Mn and Zn as EDTA chelates. The animals in the control group (C) were injected with vitamin A and E (Pro-Vit A from Virbac SA) according to the recommended dose of 1 mL/250 kg and saline at a dose of 1 mL/100 kg, and did not receive injectable selenium. The injectable trace mineral (TM) that was used in the trial is currently marketed in South Africa. These products have been used in farm animals (cattle and sheep, male and female of various ages) for years and may be considered as safe.

Milk and blood samples were collected prior to treatment (to establish baseline values) as well as at time 0, and on days 1, 2, 3, 7, 14, 30 and 60 post selenium injections and milk was also collected at 0.5 days. Blood collection was performed from the coccygeal vein or artery with the aid of evacuated tubes (vacutainer), which is a standard veterinary procedure in South Africa. The vacutainer tubes were marked according to the cow’s number. The blood-filled tubes were left to clot, the serum was then withdrawn and sent to the laboratory on ice within 12 hours after sampling. Milk collection was done by means of hand milking post teat cleaning and strip cup testing in suitable laboratory supplied collection containers then stored and sent on ice. Staff safety was constantly emphasised by investigators in collaboration with farm management.

### Laboratory analysis

The blood and milk selenium levels were measured and evaluated by the Institute for Soil, Climate and Water Laboratory, Agricultural Research Council, Pretoria, SA, using Inductively Coupled Plasma – Mass Spectrometry (ICP-MS) for selenium. Serum and milk samples were prepared for analysis by subjecting them to microwave digestion, total iodine extraction and were then left overnight, shaken for 2 h centrifuged and analysed on the same day. Samples were diluted 25-fold in the mobile phase just prior to injection and were analysed by mass spectrometry. A SC4S auto-sampler was used for total element quantification (TEQ). The iCAP Q was used in single He KED mode for measurements.

The milk composition analysis (total protein, lactose, milk urea nitrogen [MUN] and pH) was done by SA Stud Book and Animal Improvement Association using a ComboFoss Tm 7 (Rhine Ruhr).

### Data recording and analysis

Exclusion criteria: Any cow that developed clinical mastitis or a systemic disease with a fever reaction was withdrawn from the trial. Cows with milk abnormalities such as floccules, watery milk or blood in the milk as identified on the strip cup were classified as clinical mastitis and were treated accordingly. No health disorders such as milk fever, displaced abomasum and retained placenta were noted during the trial period in any of the participating cows.

### Statistical analysis

Linear Mixed Model repeated measurements analysis, also known as REML analysis, was applied to milk and serum selenium values, as well as the transformed log (base 10) SCC values to test for differences between the three treatments, the nine times (days) and the treatment by time interaction effects. *The effects over time were uncorrelated*. The residuals after analysis were acceptably normally distributed but with heterogeneous treatment variances (Freund, Mohr & Wilson 2010); therefore, *predicted* means were compared using Tukey’s test at the 1% level (Glass, Peckham & Sanders 1972) as the variances were not homogeneous. Data were analysed using the statistical programme and compared using the statistical program GenStat (VSN International 2017).

### Ethical considerations

No animals were harmed during this study. The study was conducted under field conditions using two registered drug strictly according to the recommended doses. The study was performed at the time of normal supplementation of minerals for the dairy cows in the study herd. Blood samples (collected from the coccygeal vein or artery with the aid of evacuated tubes [vacutainer]) and fore milk samples were collected at 9 occasions over a 60-day period from the cows. Sampling was done at 0 h (time 0) and days 0.5, 1, 2, 3, 7, 14, 30 and 60 during the trial period by a veterinarian.

## Results

### Milk analysis

The injectable selenium showed no effect on milk yield, lactose or MUN content of milk or its pH during this study compared with the baseline values during the trial period between the three test groups of cows. There was no indication of significant difference in the mean total milk protein between the three treatment groups of cows for the duration of this trial. There was only a numerical increase in mean total milk protein percentage over time in the group injected with Se-methionine.

### Milk somatic cell count

**FIGURE 1:**
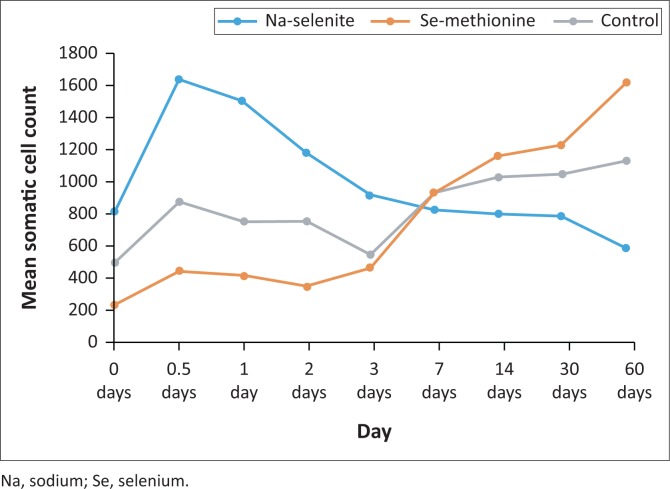
Mean somatic cell count (×10^3^ cells/mL milk) over time post selenium injection.

No significant changes were present in the log 10 SCC for both trace mineral injected groups over time. In the control group, the day 60 log 10 SCC was found to be significantly (*p* < 0.001) different from the 0 time (baseline), day 0.5 and day 3 log 10 SCC. Compared with the baseline value, the control group showed only a numerical increase in SCC from day 3 for the duration of the trial period. The SCC of the group injected with Se-methionine showed little change over the first 2 days post injection, but increased numerically from day 3 for the remainder of the trial (Figure 1). The SCC of the group injected with Na-selenite showed an initial numerical increase at day 0.5, whereafter it decreased for the remainder of the trial period (Figure 1). No correlation was evident between the log 10 SCC and the milk or serum selenium values.

### Milk selenium levels

**FIGURE 2:**
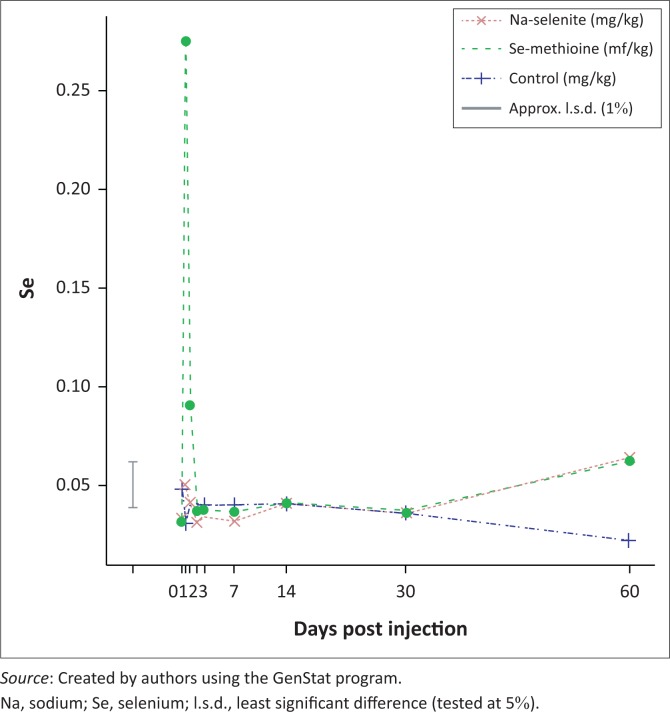
Milk selenium means per day (mg/kg) for different treatment groups.

The mean milk selenium values of the control group remained similar to the baseline values for the trial duration. The mean milk selenium concentration of the group injected with Na-selenite showed a significant (*p* < 0.001) increase at day 1 and that of the Se-methionine injected group a significant (*p* < 0.001) increase at day 0.5 and at day 1, respectively. Thereafter, there was no significant change in the milk selenium levels for the duration of the trial period (Figure 2). Compared with the control group, the milk selenium values of the group injected with Se-methionine increased significantly (*p* < 0.001) over the first day post injection of selenium (Figure 2).

### Serum selenium (mg/kg)

#### Mean serum selenium concentrations at different time intervals

**FIGURE 3:**
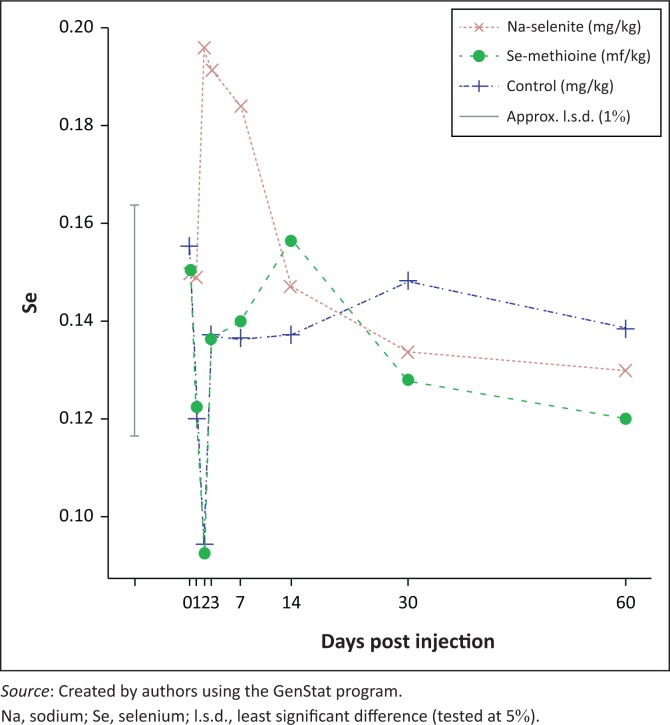
Serum selenium means per day (mg/kg) for different treatment groups.

Compared with the baseline values, the group injected with Na-selenite showed a numeric increase in mean serum selenium concentrations from day 0.5 until days 60 post selenium injection, and peaked at days 2 and 3. This increase was not significant at 1% (*p* = 0.002) but it was significant at the level 5%. The group injected with Se-methionine and the untreated control group did not show meaningful differences in mean serum selenium values compared with the baseline value (Figure 3).

The milk selenium concentrations increased compared to baseline concentrations post injection with Na-selenite and Se-methionine at day 0.5 by 51.50% and 867.00% and day 1 by 21.20% and 190.00% respectively (Table 1). The serum selenium concentrations increased on day 2 post Na-selenite injection to 104 (10%) and remained higher than the baseline concentration for the duration of the trial. Contradictory to this finding, the serum selenium concentration following Se-methionine injection remained lower than the baseline concentration for the duration of the trial period (Table 1).

**TABLE 1:** Percentage change compared with the baseline (0 time) in mean milk and serum selenium concentrations post injection of Na-selenite and Se-methionine.

Time 0 (baseline) selenium levels	Na-selenite† 0.033 mg/kg milk	Se-methionine† 0.031 mg/kg milk	Na-selenite‡ 0.096 mg/kg serum	Se-methionine‡ 0.150 mg/kg Serum
0.5 days	51.50	867.00	-	-
1 days	21.20	190.00	55.20	−18.70
2 days	18.00	19.30	104.10	−38.00
3 days	3.00	19.30	98.90	−9.00
7 days	12.00	19.70	91.60	−7.14
14 days	21.00	29.00	53.10	−8.00
30 days	12.10	19.30	40.60	−14.60
60 days	15.10	19.30	34.30	−20.00

Na, sodium; Se, selenium.

† , % Change of milk selenium levels from baseline concentration.

‡ , % Change of serum selenium levels from baseline concentration.

## Discussion

There were no specific changes identified on total milk yield, lactose and pH, compared with the baseline values, during the trial period, among the different trial groups. This corresponds with research indicating that injectable selenium shows no effect on milk production, protein or lactose (Ceballos-Marques et al. 2012; Machado et al. 2013).

Milk composition (butterfat and total protein) is influenced by breed, genetics, environment, udder health, physiological stage of lactation and nutrition. In addition, other factors such as rumen buffers and mineral intake also play a role. Research has demonstrated that milk protein, besides other factors, is being influenced by amino acid nutrition (Hosam, Azzam & Alinimer 2013). Compared with the milk protein or the amino acid profile of rumen microbes, feeds are often deficient in methionine contents. The protein that reaches the small intestine is adequate in cysteine but deficient in methionine, making it the first limiting amino acid for high-yielding dairy cows. Research shows inconsistent results concerning the effects of methionine supplementation on milk protein (Hosam, Azzam & Alinimer 2013). Diets low in rumen-degradable protein or balanced for optimal microbial protein synthesis should increase supplies of amino acids available to the mammary gland to be used for protein synthesis (Linn 1988). Abomasal infusions of amino acid mixtures also increased milk protein percentage, with methionine and lysine accounting for more than 68% of the observed increase. Based on these responses, it could be concluded that by increasing the intestinal supply of amino acids through increased rumen protein synthesis or low rumen-degradable protein sources such as rumen protected methionine (Kruze et al. 2007), would increase milk protein percentage. Research however, showed inconsistent results regarding the effects of oral methionine (Richeson et al. 2006) as well as injectable selenium on milk protein (Machado et al. 2013).

An increase in serum selenium concentration occurred in this trial following Na-selenite injection from 0.5 to 14 days. This finding correlated with other research that indicated peak selenium concentration in blood and serum 5 h post intramuscular injection of selenium that lasted for 28 days (Arthington et al. 2014). No significant differences in average serum selenium values post injection with Se-methionine were indicated in this trial, which was similar to findings of Pechova et al. (2008) and Davidov et al. (2014). However, these findings differ from those of Prased and Aora (1991) that indicated an increase in blood selenium levels following orally supplemented Se-methionine. Grace et al. (2001) indicated a relationship between blood selenium (GSH-Px) and milk selenium concentrations post injection of barium selenite (Ba SeO_4_).

Ganda et al. (2016) indicated that injectable trace mineral tends to reduce the proportion of cows diagnosed with subclinical mastitis and the cure of subclinical mastitis cases was associated with higher serum concentrations of phosphorus and selenium. Initial reduction in SCC was reported by Oltramari et al. (2014) following oral supplementation of selenium of dairy cows, but showed evidence of a significant decrease in SCC after 5 days (Mc Dowell et al. 2005). Oltramari et al. (2014) indicated that cows subjected to oral organic selenium supplementation presented a lower SCC than those subjected to inorganic selenium supplementation. This could not be confirmed with the injectable Se-methionine supplementation during this trial.

Although the decrease in average SCC is not significantly lower for every time interval tested, the constant numerical decrease following Na-selenite injections could be indicative of improved immunity in the udder. The drop of the average SCC to levels lower than the baseline cell counts on day 60 post injection is suggested to improve udder health following the injection of inorganic Na-selenite as selenium source. The injection of Na-selenite also led to a negative correlation between average milk selenium concentration and average SCC over the 60-day trial period. This finding corresponds with literature stating that bulk tank milk selenium concentration responds rapidly to changes in selenium intake (Ceballos-Marques et al. 2012; Davidov et al. 2014), but the results do not correlate with research done by Alejandro et al. (2012), which indicated that there was no relationship between bulk tank selenium concentrations and SCC. The untreated control cows showed a constant percentage increase in average SCC compared with the groups of cows that were injected with Na-selenite for the duration of the trial.

For the first 3 days after administering selenium, the Se-methionine supplemented group of cows showed a higher percentage decrease in average SCC compared with the baseline value than the Na-selenite and control groups. However, from day 3 until the end of the trial period, there was a steady increase in average SCC for this group. Because trace minerals play an important role in immunity and udder health, it could be speculated that the highly significant excretion of selenium via the milk over the first day of supplementation as an organic Se-methionine compound could have led to the inconstant decrease in average SCC. It could also be speculated that the excretion rate of the selenium from the milk over the first day could have resulted in a positive correlation between average milk selenium concentrations and average SCC from day 3 post injection.

The extent of the increase in milk selenium concentration after administering Se-methionine compared with the milk selenium concentration after Na-selenite injection correlated with research done by Juniper and Bertin (2006). The milk selenium content was found to be directly correlated with the organic selenium content in the feed of cows (Mehdi & Dufrasne 2016). Ortman and Pehson (1999) indicated that organic selenium was more effective than inorganic selenium in increasing the milk selenium concentration. According to Slavik et al. (2008), the vast majority of selenium in milk, when Se-yeast is fed, is in the form of Se-methionine. Cows supplemented with organic selenium showed an increase in milk selenium concentration of up to 190% higher than cows supplemented with inorganic selenium (Mehdi & Dufrasne 2016; Ortman & Pehrson 1999). Both these previous findings correlated with the findings of injectable organic Se-methionine and inorganic Na-selenite used during this trial.

Treatment effects, such as changes in milk selenium concentrations post selenium injection, indicated improved bioavailability of selenium from the supplemented source (Juniper & Bertin 2006). At the recommended dose rate of oral selenium supplementation (0.3 mg/kg), replacing selenite with organic selenium will almost double the bulk tank selenium, while increasing plasma or serum selenium concentrations by 10% – 20% (Ceballos-Marques et al. 2012). Research has shown that repeated injections with Se-methionine can increase the milk selenium levels as much as 27- to 54-fold compared with no treatment from as early as day 7–15 for up to 95 days (Patent 2010). During this trial, the milk selenium values were increased 10-fold within 12 h post injection. The reason for the earlier and lower peak of average milk selenium concentrations post injection could be that this Se-methionine was injected as a single dose with no trace mineral supplementation prior to that.

Milk, as a significant component of human diets, can provide a high proportion of the total selenium intake - that is, 100 g of milk can provide up to 10% of the daily selenium requirements for adults (Cobo-Angel, Wichtel & Ceballos-Marques 2012). The selenium concentration of milk is easily manipulated, and this can be a useful tool for increasing selenium intake for humans, especially when organic sources of dietary selenium are preferred over inorganic sources (Cobo-Angel et al. 2012). An oral intake of approximately of 25 mg selenium per day from Se-yeast (Se-methionine) may however produce milk that exceeds the legal limit of selenium concentrations of 0.14 mg/L for human consumption (Weiss 2010). The Se-methionine injection during this trial leads to milk with an average milk selenium concentration of 0.3 mg/kg within 12 h post injection. This selenium level exceeds the toxic levels for human consumption, according to the FDA of the United States, Department of Health and Human Services, which is most probably due to the nature and the metabolism of the amino acid binding of the selenium source. Se-methionine is transferred to milk better than inorganic selenium sources such as Na-selenite. This more effective transfer could be explained by the greater methionine concentration in milk because Se-methionine originating from Se-yeast is readily incorporated into milk (Cobo-Angel et al. 2012). It is important to investigate selenium concentrations in bulk milk for human consumption, especially if all the production animals were simultaneously injected, which is the case in many dairies in South Africa. People with adequate levels of selenium should not use selenium supplements because studies indicate that too-high selenium concentrations may be associated with Type 2 diabetes in well-nourished populations (Cobo-Angel et al. 2012). Further studies are needed regarding this result. It may be necessary to impose a withdraw period on milk for human consumption post injection of lactating dairy cows with Se-methionine.

Research shows that selenium supplemented orally in the form of Na-selenite would have to be fed at toxic levels to the cow before there would be any measurable increase of selenium in milk (Fisher, Hoogendoorn & Montemurro 1980), which correlates with the results of the injectable Na-selenite in this trial. Previous research by Davidov et al. (2014) indicated a negative correlation between serum selenium concentrations and SCC. High serum selenium concentrations were associated with reduced rates of mastitis and lower bulk tank SCC in Ohio dairy herds (Ganda et al. 2016; Shen et al. 1999). The results from this trial correspond with previous research by indicating a negative correlation between average serum selenium values and SCC post injection of Na-selenite for a period of 60 days. In contrast to orally supplemented trace mineral supplements, which were included at higher concentrations in the daily ration and supplemented over a long period of time, injectable trace mineral supplements are administered at lower concentrations. Their administration can also be targeted 3–4 times a year at critical physiological stages of animals to improve production, reproduction and immunity. The injectable inorganic Na-selenite showed a similar response to oral organic selenium supplementation during this study. Injectable Se-methionine only showed corresponding results with previous oral supplementation of Se-methionine regarding butterfat and milk protein percentage, and for a limited time on both average milk and serum selenium concentrations indicating a negative correlation with average SCC.

## Conclusion

Selenium supplementation is used strategically during physiological periods to improve milk production, reproduction and immunity in South African dairy herds. During the initial 72 h following injection with the organic selenium (Se-methionine), the milk selenium levels increased 10-fold and a negative correlation was present between milk selenium levels and SCC. However, blood selenium levels remained unchanged. The high milk selenium levels may exceed the legal limit of selenium concentrations of 0.14 mg/L for human consumption and warrant further investigation. Following injection with the inorganic selenium (Na-selenite), blood selenium levels stay increased for a 14-day period and milk selenium increased significantly for 24 h but not to the same levels as was noted after treatment with the injectable organic selenium (Se-methionine). No negative correlation was present between milk selenium and the somatic cell counts in the milk in treated cows. The strategic injection Na-selenite, as a source of selenium in the formulation of Multimin, showed a high potential benefit for the farmer as evaluated during this trial period. Further studies are needed to evaluate the possible benefit of the injectable trace mineral’s use in combination with daily oral trace mineral nutrition.
